# A solar panel dataset of very high resolution satellite imagery to support the Sustainable Development Goals

**DOI:** 10.1038/s41597-023-02539-8

**Published:** 2023-09-20

**Authors:** Cecilia N. Clark, Fabio Pacifici

**Affiliations:** Maxar Technologies, Maxar Labs, Westminster, CO 80234 USA

**Keywords:** Energy infrastructure, Photovoltaics, Sustainability

## Abstract

Effectively supporting the United Nations’ Sustainable Development Goals requires reliable, substantial, and timely data. For solar panel installation monitoring, where accurate reporting is crucial in tracking green energy production and sustainable energy access, official and regulated documentation remains inconsistent. Reports of solar panel installations have been supplemented with object detection models developed and used on openly available aerial imagery, a type of imagery collected by aircraft or drones and limited by cost, extent, and geographic location. We address these limitations by providing a solar panel dataset derived from 31 cm resolution satellite imagery to support rapid and accurate detection at regional and international scales. We also include complementary satellite imagery at 15.5 cm resolution with the aim of further improving solar panel detection accuracy. The dataset of 2,542 annotated solar panels may be used independently to develop detection models uniquely applicable to satellite imagery or in conjunction with existing solar panel aerial imagery datasets to support generalized detection models.

## Background & Summary

Advancements in remote sensing data acquisition and processing support novel capabilities for collecting valuable information in satellite imagery, providing prompt and comprehensive data from local to global scales^1,2^. Small target detection has become particularly useful in addressing several of the United Nations’ Sustainable Development Goals (SDGs) by supplementing traditional methods including ground surveys, official statistics, and limited aerial observations^1,3,4^. Small targets have historically been difficult to accurately identify with meter-scale resolution satellite imagery, but advancements in satellite technology, image processing, and dissemination of higher resolution imagery in publicly available datasets has shifted this narrative^4–6^.

One such use case which may benefit from very high resolution (VHR), or sub-meter, satellite imagery is solar panel detection and monitoring to support SDG 7, which addresses affordable and clean energy, and SDG 13, a goal focusing on actions to combat climate change. As the use of renewable energy becomes more widely adopted, there should exist regulated reporting and assessment measurements to best inform policy-making both regionally and globally^7^. However, for residential solar panel installations, there are varying documentation requirements dependent on region and installation provider^7,8^. Developing accurate solar panel detection models using remote sensing data will complement typical reporting methods, with satellite imagery proving specifically useful for frequent observations with global coverage^5^. With increasingly reliable reporting of residential solar panel installations, researchers and policy-makers can make more informed decisions regarding monitoring and rectifying existing limitations to residential-scale renewable energy access and implement data-driven solutions to combat climate change.

Residential solar panels are considered small, weak targets even in VHR satellite imagery due to the average number of pixels per object, variation among objects, and complex context^4^. Existing satellite imagery datasets often include large-scale, or non-residential, solar panel annotations due to resolution of the imagery and therefore ability to detect small objects^9,10^. There are available datasets of VHR imagery to support accurate detection of small-scale and residential installations, but the imagery is generally sourced from aerial platforms^8,11–19^. These collection sources limit object detection models trained on such datasets to the same types of imagery for testing due to variability in visible objects, illumination, angle, atmospheric visibility, and sensor characteristics between aerial and satellite images. Thus, rather than rapidly acquiring and processing large coverage data, researchers and policy-makers are limited to local and potentially outdated aerial imagery for monitoring and reporting.

To address these limitations, we provide a VHR satellite imagery dataset of annotated, primarily residential, solar panels to supplement the ever-growing list of solar panel datasets. This dataset may be used independently or in conjunction with larger, non-satellite imagery datasets to produce robust detection models capable of generalizing across image types. The dataset may be used as provided or with additional variations in image conditions, implementations of cross validation across images and image subsets, or modifications to the ratio of annotated panels across confidence levels, as described in the “Technical Validation” and “Usage Notes” sections. Using any portion of this dataset toward solar panel detection applications may better support the use of satellite imagery in rapidly detecting and monitoring residential-scale solar panel installations, allowing researchers and policy-makers to address the needs of various applications for global, timely, and consistent renewable energy monitoring and reporting.

**Fig. 1:**
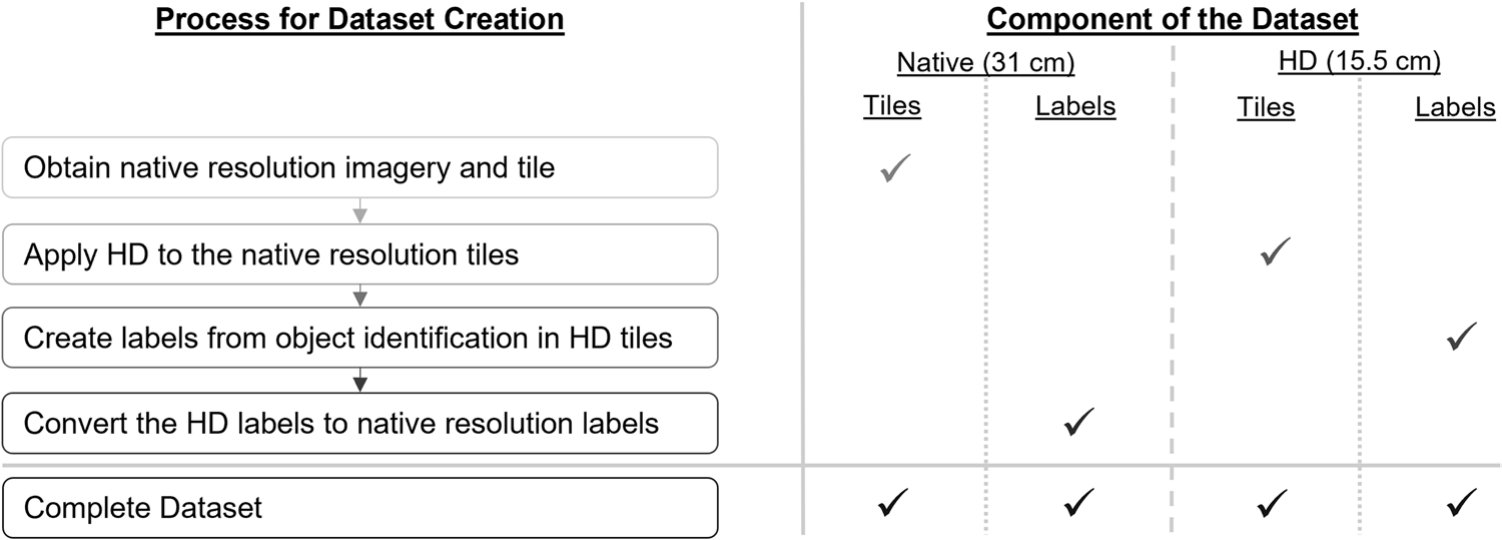
The process for creating the paired native resolution and HD image tiles and associated labels. Both sets of components contain three image tiles and 2,542 annotated solar panels.

We also expand upon the results of current publications investigating the resolution-performance trade-off for accurate small object detection in satellite imagery^20–25^. Results of these studies indicate accuracy of small object detection in satellite imagery may be improved by increasing spatial resolution. Therefore, our dataset contains 31 cm native resolution satellite imagery paired with Maxar Technologies’ High-Definition (HD) 15.5 cm resolution imagery, which could represent what a space-borne sensor would acquire at this resolution. The inclusion of HD imagery supports studies examining the effects of spatial resolution on small, sustainability-motivated object detection. The complete dataset contains native resolution satellite imagery, corresponding HD imagery, and solar panel object labels for each image type (Fig. 1). To the best knowledge of the authors, there are no publicly available datasets including annotated solar panels in native resolution and HD satellite imagery.

The native resolution and HD images were cropped proportionately to alleviate memory constraints and prevent image resampling when input into object detection models. The resulting image chips and labels are provided in a format compatible with the You Only Look Twice version 4 (YOLTv4) architecture^26^. YOLTv4 is an object detection framework developed to address the issue of small object detection in satellite imagery and is openly available (https://github.com/avanetten/yoltv4). We are providing the dataset open-source in a YOLTv4-suitable format to support goals of data philanthropy and accessibility.

While the dataset is limited in variety of landscapes and image conditions, the value provided by this dataset may benefit research regarding:

- identification of small-scale solar panels in satellite imagery to monitor green energy production and sustainable energy access,
- detection of small, sustainability-motivated objects in VHR satellite imagery, and
- investigation into the effects of spatial resolution in remote sensing data analysis.

## Methods

### Image acquisition

One image over southern Germany was acquired from WorldView-3, a 30 cm-class Maxar Technologies satellite. Southern Germany was selected as the area of interest due to the high concentration of solar panels, both residential and commercial, reported in the region^27,28^. Images over the area of interest were inspected for characteristics to minimize overhead occlusion, variation in solar panel angles and orientations, and potential glare. The final image, Catalogue ID 1040050029DC8C00, met these criteria with 0.0% cloud coverage, 3.9° off-nadir angle, and 42.2° sun elevation. The image was originally collected on September 18, 2020 and has a maximum Ground Sampling Distance (GSD) of 31 cm.

**Fig. 2:**
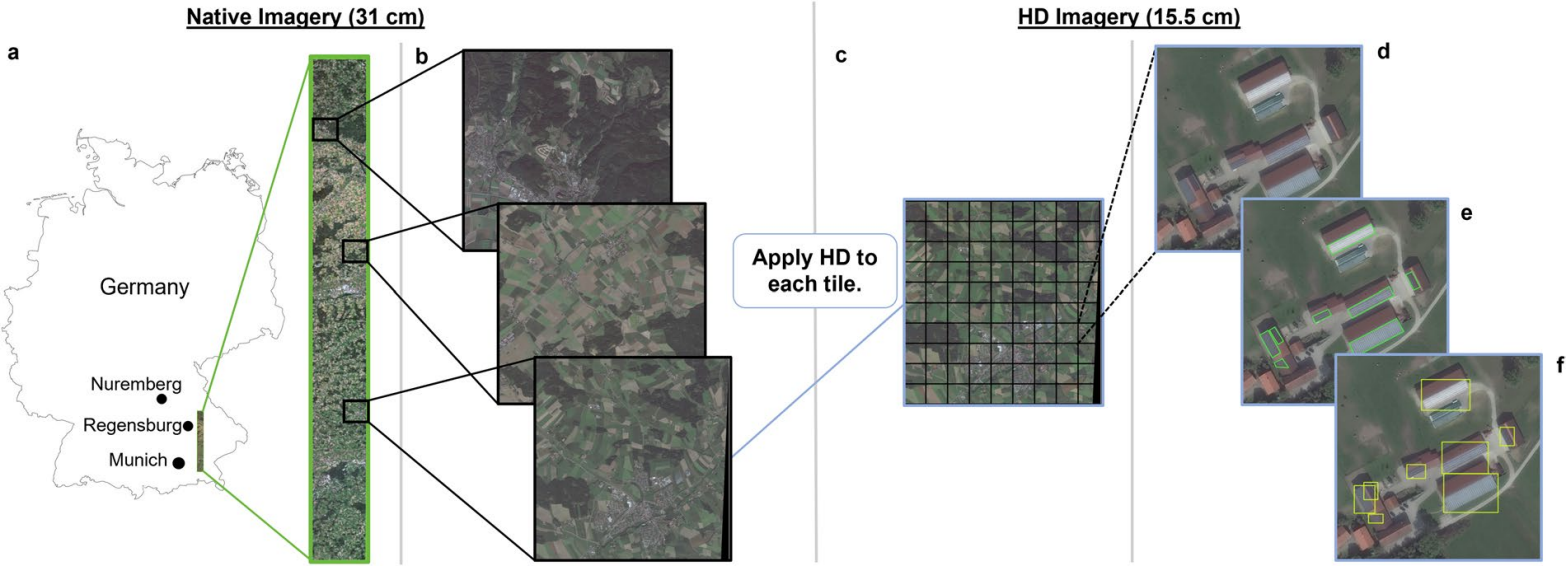
The data partitioning and annotation process. (**a**) The location of the full image in southern Germany, where the full native resolution image is outlined in green. (**b**) The locations of the selected tiles from the native resolution image. The native resolution tiles are outlined in black. (**c**) A representation of the manual scanning of each tile after HD has been applied. The HD tile and subsequent labeling windows are outlined in blue. (**d**) The first step of the annotation process: identify solar panel objects in the scanning window. The dotted lines indicate the image shown is only a portion of the annotation window in the full HD tile. (**e**) The second step of the annotation process: create polygons around each identified object, labeled as green polygons. (**f**) The third step of the annotation process: convert polygons to individual horizontal bounding boxes, labeled in yellow.

The full native resolution image was obtained along with corresponding tiles, which have a default size of 16,384 by 16,384 pixels (approximately 5 km by 5 km projected onto the ground). Three tiles were randomly selected from the entire image and used for dataset creation (Fig. 2). Two tiles were standard size, and the third tile was 15,181 by 16,384 pixels (approximately 4.7 km by 5 km projected onto the ground) due to the size and shape of the original image, resulting in irregular tiling in certain regions.

The three HD resolution tiles were derived from the three native resolution tiles, resulting in a second set of imagery with four times as many pixels as the original tiles. Two of the HD tiles were 32,768 by 32,768 pixels in size and one was 30,362 by 32,768 pixels in size. The GSD of each tile was not changed, but the resulting spatial resolution of the HD tiles is 15.5 cm. The visual information between both sets of imagery is similar, resulting in comparable image pairs.

### Defining solar panel objects

Solar panel objects were labeled using Quantum Geographic Information System (QGIS) software (https://qgis.org/en/site/) version 3.20.0 ‘Odense’ when identified in the HD tiles, in which the solar panel objects were easier to visualize and distinguish from other objects of similar size and shape. Individual solar panels were not labeled, as unique objects of that size are difficult to distinguish even at 15.5 cm resolution. Instead, groups of solar panels, or solar panel arrays, were labeled and counted as a single “solar panel.” Panel objects were labeled when visible distinctions could be made between two or more objects, determined by varying size, shape, and/or space between them.

**Fig. 3:**
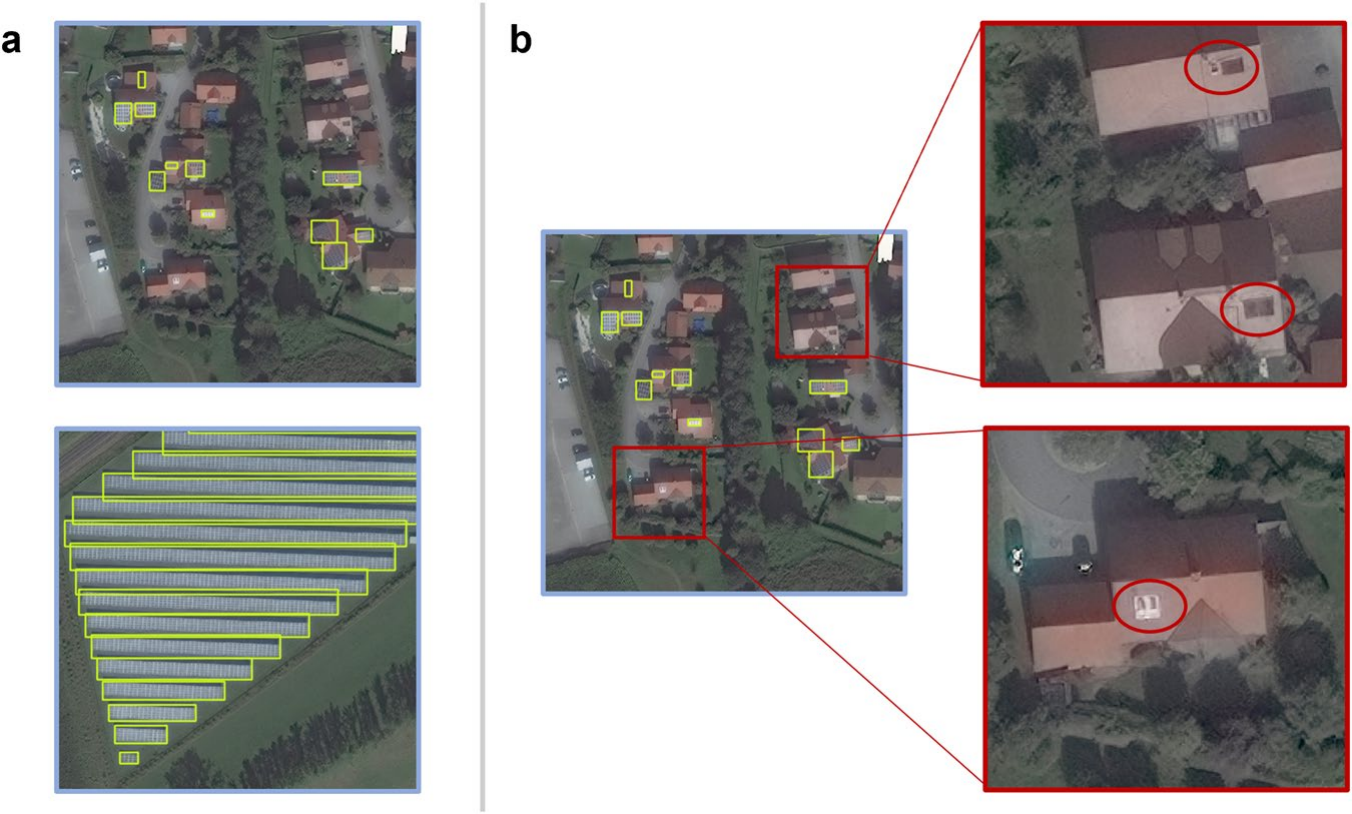
Examples of solar panel objects and non-solar panel objects. (**a**) Single solar panels in residential areas were labeled with a unique bounding box, labeled in yellow, where individual panels were determined by differences in size, shape, and spacing (top). For large ground-mounted panels, one label constitutes one row (bottom). The HD labeling windows illustrating confirmed panels are outlined in blue. (**b**) Objects on residential roofs with less than three distinct panel shapes did not meet the necessary criteria to be confidently identified as solar panels and were treated as non-solar panel objects. The windows illustrating non-solar panel objects are outlined in red, with specific non-solar panel objects circled in red.

For roof-mounted solar panels, single objects with at least three distinct components, considered to be three identifiable and unique panels, were identified as solar panel objects. Objects with potentially only one or two panels, either adjoined or separated, were treated as non-panel objects such as skylights, ventilation caps, and chimneys (Fig. 3). For large ground-mounted panels, specifically those in solar farms, solar panel objects were identified by spacing. These objects were often uniform in shape and varied slightly only in size. Individual objects were therefore labeled based on distance between them, resulting in rows of solar panels being labeled as individual objects (Fig. 3).

### Annotating solar panel objects

For each HD tile, a single tile was added as a raster layer into QGIS. A shapefile layer was added overlaying the HD tile, to which solar panel objects were recorded. The three HD tiles were visually scanned using an approximate 250 m by 150 m window at a scale of 1:700 (Fig. 2).

During the visual scan of each tile, a polygon feature was created for each identified solar panel object and contained the entire object. After scanning the full HD tile, the polygon features were separated from one multi-polygon label to single polygons, and each single polygon was then converted to a horizontal bounding box (Fig. 2). In total, 2,542 solar panel objects were labeled with unique horizontal bounding boxes across all three HD tiles.

The shapefile containing the bounding box pixel coordinates for each of the HD tiles was exported as a unique GeoJSON file. Bounding box coordinates for the native resolution tiles were obtained by dividing the HD tile coordinates by a factor of two, determined by the relationship between the HD and native images: HD tiles are twice the size of native resolution tiles in both x and y directions. The bounding box coordinates for the native resolution tiles were verified through visual inspection.

### Creating image chips and labels

Image and label chips were created from the two sets of tiles and their corresponding labels. To maintain general consistency in visual information, the native tiles were chipped to size 416 by 416 pixels and the HD tiles were chipped to size 832 by 832 pixels. Chips were created as input for the YOLTv4 architecture^26^, where one solar panel object is centered in a single chip, resulting in 2,542 native resolution chips with corresponding labels and 2,542 HD chips with corresponding labels. Due to the close proximity of many solar panels objects, most chips contain more than one labeled object.

## Data Records

**Fig. 4:**
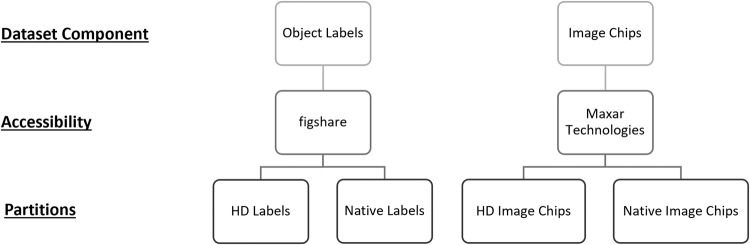
The file structure for the complete dataset as described in the “Data Records” section^29,30^. All partitions contain 2,542 files each, with object labels and image chips corresponding by image type (native or HD).

The dataset consists of two parts: one containing object labels and another containing image chips (Fig. 4). The object labels, “Solar Panels in Satellite Imagery: Object Labels,” are provided as text files and can be accessed on *figshare* (10.6084/m9.figshare.22081091)^29^. The image chips, “Solar Panels in Satellite Imagery: Image Chips,” are available directly from Maxar Technologies as TIF files (https://resources.maxar.com/product-samples/15-cm-hd-and-30-cm-view-ready-solar-panels-germany)^30^. Both object labels and image chips include native and HD components.

The label files available on *figshare* contain information for each solar panel object, at both native and HD resolution, in the form of center points, length, and width of each bounding box, all normalized by chip size. Specifically, each label contains at least one row of information, where a single row represents a unique solar panel object with the structure: category, x-center, y-center, x-width, y-width. The category for each solar panel object is 0 for objects identified with high confidence, 1 for objects identified with moderate confidence, and 2 for objects identified with low confidence. Determining the level of confidence for each solar panel object is described in the “Technical Validation” section.

The image chips can be accessed following the link provided in the “References” section and with the object labels, which will direct users to a Maxar Technologies’ access download page. Data access requires user name, email address, affiliation, and country. The image chips are provided as individual TIF files and follow the naming convention of the corresponding labels. The 2,542 native image chips are 416 by 416 pixels in size, and the 2,542 HD image chips are 832 by 832 pixels in size.

The naming convention for all labels and image chips includes the name of the dataset, image type, tile identification number, minimum x bound, minimum y bound, and window size. The minimum bounds correspond to the origin of the chip in the full tile. An example image chip would be “solarpanels_hd_1__x0_0_y0_14027_dxdy_832.tif,” which is an HD chip from Tile 1 in the solar panel dataset, with a minimum x bound of 0, minimum y bound of 14,207 and window size of 832 by 832 pixels. The corresponding label file for this example would be “solarpanels_hd_1__x0_0_y0_14027_dxdy_832.txt.”

## Technical Validation

To create the satellite imagery dataset, individual solar panel objects were manually annotated. While this method can be thorough and consistent when executed efficiently, manual annotation is tedious. Even under very rigorous conditions, annotators are prone to mistakes, which can result in unidentified and misidentified solar panel objects^17^. Additionally, the shape of each label may incorrectly encompass a single object.

Unidentified and misidentified solar panel objects can be caused by poor image resolution resulting in the objects being difficult to distinguish from the background, inconsistent definitions of what defines a solar panel object resulting in erratically labeled objects, and insufficient amount of time spent annotating each tile. We took the following precautions and approaches to address these sources of error during labeling:

- *Poor Image Resolution:* The HD tiles were used during the labeling process rather than the native resolution tiles. The increased detail in the HD tiles supported pronounced distinction between objects and the background (differentiation between a solar panel and markings on a roof) as well as within each object (differentiation between a solar panel or glass roofing).
- *Inconsistent Definitions:* A list of solar panel object identifiers, as mentioned in the “Methods” section, was provided and consulted throughout the labeling process. When a solar panel object could not be immediately confirmed, the list of identifying features was used as a benchmark to either confirm or reject the object in question as a solar panel object. Implementing the definition as a checklist minimized inconsistencies throughout identification. Homogeneity in the annotation process was additionally supported by using one annotator to label the full dataset.
- *Cursory Labeling:* Manual annotation may become monotonous over prolonged periods of time, resulting in rushed and potentially incorrect labels. To minimize the error presented by this issue compounded with seeing repetitive areas in a short amount of time, a single annotator examined each tile at least twice, with no less than one week between examinations. This method was designed to minimize incorrect identification in visually-redundant and high-concentration areas.

The shape of each solar panel object was another potential source of error as a consequence of manual annotation. To minimize errors caused by the solar panel object shape, and to conform with the necessary input shape for the YOLTv4 model, each polygon shape was converted to a single horizontal bounding box. Horizontal bounding boxes generally do not compactly encompass an object, and more background pixels are included in the final object. Most errors caused by any irregular shape of the polygon feature have less influence with this method.

### Confidence of solar panel identification

Official maps of existing solar panels in a given region are difficult to obtain as a result of multiple installation providers supplying the area of interest and sporadic public indexing installation information^8,13^. However, there does exist a baseline “map” of visible solar panels available in Google Earth (https://earth.google.com/). When looking at the selected regions of southern Germany in Google Earth, the image resolution is higher than the imagery available in the solar panel dataset. The Google Earth imagery was collected from a non-satellite aerial sensor, and the improved resolution provides greater visible definition in each solar panel object. The centimeter-scale resolution supports the use of Google Earth imagery as a reliable “ground truth.”

Using the coordinate search function in Google Earth, version 9.175.0.1, the location of each solar panel object in the dataset was identified and examined for the existence of a solar panel or similar object. The level of confidence at which a solar panel object could be, or not be, identified was added to the label for that specific object. The levels of confidence and associated category labels are described as:

- *Category 0, High Confidence:* The solar panel object labeled in the dataset was identified and confirmed as a solar panel at the same location in Google Earth.
- *Category 1, Moderate Confidence:* The solar panel object labeled in the dataset could not be identified and confirmed as a solar panel at the same location in Google Earth. The labeled solar panel object could not be misidentified as another object at the same location in Google Earth. The labeled solar panel object met all of the identifying criteria defining a solar panel.
- *Category 2, Low Confidence:* The solar panel object labeled in the dataset could not be identified and confirmed as a solar panel at the same location in Google Earth. The labeled solar panel object could be misidentified as another object at the same location in Google Earth.

The highest factor contributing to Category 1 solar panel objects was a lack of building or panel construction between the time of dataset image acquisition and the reported date of the imagery provided over the region in Google Earth. This relationship is shown in Table 1, where the dataset image acquisition date (September 18, 2020) can be compared to the reporting dates of the Google Earth verification images. For dates before the dataset image acquisition date, the number of unconfirmed panels is greater due to the lack of either buildings constructed or solar panels installed within that time. For Tile 3, with a date after the dataset image acquisition date, the number of unconfirmed panels is smallest and consists primarily of solar panel objects that could not be confidently verified as solar panels (Category 2). The most common errors contributing to Category 2 objects are glass and/or metal roofing misidentified as solar panels.

**Table 1:** Summary of the number of solar panel objects in each image tile and the proportion of confirmed (high confidence) objects.

Tile	Verification Date	High Confidence	Moderate Confidence	Low Confidence	Total Objects (% Confirmed)
Tile 1	June 30, 2019	966	1	22	989 (97.7%)
Tile 2	March 16, 2020	617	19	2	638 (96.7%)
Tile 3	June 15, 2021	904	2	9	915 (98.8%)
**Total**	—	**2,487**	**22**	**33**	**2,542 (97.8%)**

Individual image tiles listed here represent both the HD and native resolution components, as labels are consistent across image sets. The reported date is provided for each verification image, determined by the date listed over that region in Google Earth during the time validation was conducted. The reported verification date may be compared to the satellite image collection date (September 18, 2020).

All tiles in the solar panel dataset were compared to the Google Earth verification imagery, and all objects were categorized accordingly. Category labels were kept consistent across the native and HD tiles. In total, 2,487 solar panel objects, or 97.8% of the final dataset for each image type, were identified with high confidence. Less than 3.0% of the solar panel objects were identified with moderate or low confidence.

## Usage Notes

The image chips are provided as TIF files. The labels for each image chip are provided in text format, and the name for each label matches the name of the corresponding image chip. Each row of a label file contains the category and bounding box coordinates for a single solar panel object, as described in the “Methods” section. To avoid the need for re-projection of the object coordinates before use, the solar panel object coordinates are provided in pixel coordinates for the corresponding image chip. The pixel coordinates are provided as normalized center points along with width and height of each solar panel object in the associated chip.

While the image chips do provide valuable satellite imagery, they are limited in variety to a single set of image conditions based on the nature of the original image acquisition. If researchers wish to use the annotations to identify solar panel objects in a variety of image conditions (i.e., differences in illumination and look-angles) over the same geographic region, the pixel coordinates for each labeled object may be translated to geocoordinates using the information and instructions listed with the labels in *figshare*^29^. The confidence of each object label in any imagery not currently provided as the associated image chips would need to be reassessed.

The naming convention of the image chips and corresponding labels allows users to select a subset of any single image, a combination of images, or withhold one image from training to use in testing. For coordinate conversion, the naming convention also indicates the associated tile from which each label originated, where each tile has a unique geotransform. The category labeling of each panel also allows users to remove any unconfirmed solar panels (those labeled with moderate and low confidence) from the dataset and only use confirmed objects (those labeled with high confidence).

### Attributing satellite imagery

Any use of the Maxar Technologies images must include the following statement: “© 2023 Maxar Technologies. This imagery is provided under the Creative Commons Attribution-NonCommercial 4.0 International Public License (https://creativecommons.org/licenses/by-nc/4.0/legalcode#s3a1Ai).”

## Data Availability

The native resolution images were provided in tile format (either 16,384 by 16,384 pixels or 15,181 by 16,384 pixels in size) by Maxar Technologies. Creating the HD tiles from the native resolution tiles was accomplished with the use of Maxar Technologies’ proprietary HD technology. Analyzing the HD image tiles and labeling individual solar panel objects was completed using QGIS version 3.20.0-Odense. QGIS is a free, open-source software and supports object labeling through various methods. Labeling for this dataset was completed using the “Add Polygon Feature,” “Multipart to Single Parts,” and “Bounding Boxes” tools, where the output of each component was the input of the next. The final GeoJSON file for each tile was obtained by exporting the output of the “Bounding Boxes” tool. Converting the image tiles and associated label GeoJSON files to image chips and associated label text files was completed using open-source code provided with the YOLTv4 architecture^26^. Documentation for additional processing of the image chips and labels, as well as for training a YOLTv4 model using a dataset structured this way, can be found in the GitHub repository referenced in the “Background & Summary” section.
